# New failure mechanism for evaluating seismic and static undrained bearing capacity adjacent to 2D slope

**DOI:** 10.1038/s41598-023-28415-4

**Published:** 2023-01-31

**Authors:** Hongwei Fang, Yixiang Xu

**Affiliations:** 1 School of Geomatics and Prospecting Engineering, Jilin Jianzhu University, Changchun, 130118 China; 2 School of Aerospace, UNNC-NFTZ Blockchain Laboratory, University of Nottingham Ningbo, Ningbo, 315100 China

**Keywords:** Natural hazards, Solid Earth sciences

## Abstract

The seismic or static undrained slip line field theory and Cauchy, Riemann, mixed boundary value problems for undrained soil slopes are derived. A new failure mechanism is proposed to determine the undrained bearing capacity adjacent to slopes. The effects of geometric or strength parameters and seismic forces on static and seismic undrained bearing capacity are investigated. The convergence of the proposed method is proved. The static and seismic undrained bearing capacities predicted by the proposed method are close to those of the currently existing methods. The proposed method does not need to assume or search the failure modes, and a new limit state evaluation index is given.

## Introduction

The retaining structures, bridge abutments, and transmission towers adjacent to a slope generally weaken the stability of foundations. How to determine the ultimate bearing capacity is the primary problem when a foundation is adjacent to a cohesive slope. The widely used methods determining the undrained bearing capacity adjacent to slope are limit equilibrium method (LEM)^1^, limit analysis (LA)^2^, slip line field theory (i.e., the method of characteristics)^3^, finite element method (FEM)^4,5^, lower bound (LB), and upper bound (UB)—finite element limit analysis (FELA)^6^. For a foundation-on-slope system, the ultimate bearing capacity of a cohesive slope is determined by the combination of foundation failure and global slope failure, which is difficult to be predicted using traditional methods^7^. Thus, the failure mechanism is the key problem, which includes two issues: one is to determine the location and shape of the critical failure surface (i.e., the failure models), and the other is the choice of the instability criterion, i.e., how to judge whether the slope is in a limit state.

At present, the first issue is solved by hypothesis and optimization search. For example, LEM assumes a circular failure mechanism; the symmetrical and nonsymmetrical failure mechanisms are considered in the UB of the limit analysis^8^; the UB-FELA uses discontinuous quadratic displacement fields and second-order cone programming to obtain the clear failure mechanism of the computational domain^9^; the broken line random failure mechanism was used in UB of the limit analysis^10^; the discontinuity layout optimization (DLO) uses LB and UB as the limit state plasticity failure discretization scheme^11^.

However, the failure model is subjected to change due to the influences of strength parameters (i.e., soil characteristics), geometrical parameters (i.e., slope angle, slope height, foundation width), and seismic acceleration. Five seismic and static models, i.e., slope face failure, below-toe failure, two bearing capacity failure, and overall slope failure, were obtained using FEM^4,5^ and UB plasticity solution^2,12^. Thus, the failure model is challenging to be identified in advance for a specific case^13^.

For the second issue, the factor of safety (FS) is adopted to conduct the limit conditions, i.e., the slope is in a limit state when FS = 1. Numerical criteria, such as the non-convergence criterion for computation and the mutation criterion for displacements, are also used to investigate slope stability in numerical simulation. The disadvantage of numerical criteria requires subjective interpretations.

The slip line field theory has been widely used in the calculation of the ultimate bearing capacity of plane foundation^14^. The current slip line field theory considers the outermost slip line as the critical slip surface to predict the bearing capacity adjacent to slope^15^. However, each slip line may be a slip surface based on the Mohr–Coulomb criterion. Only the slip line associated with the minimum safety factor can be regarded as a critical slip surface. The slope under the limit state condition, i.e., critical slope contour, can be calculated by the slip line field theory. Fang et al.^16^ proposed a new instability criterion, which is only applicable when the cohesion is not zero, and the critical slope contour intersects with the slope bottom. The static and seismic slip line field theory of the pure cohesive soil slope is derived using the method of characteristics. Based on the pseudo-static method, a new failure mechanism is proposed for evaluating the seismic and static undrained bearing capacity of shallow strip foundations adjacent to the pure cohesive soil slope. The influence rule of geometrical or strength parameters and seismic force on the undrained bearing capacity are discussed to validate the feasibility of the proposed failure mechanism. According to the distribution, the load on the top of the slope can be divided into three categories: point load, line load, and surface load. The proposed method is mainly applied to the surface load.

## Approach

### Governing equations

According to the Mohr–Coulomb criterion, the expressions of normal stress and shear stress are:

1a $$\sigma_{x} = \sigma (1 + \sin \phi \cdot \cos 2\theta ) - c \cdot \cot \phi$$

1b $$\sigma_{y} = \sigma (1 - \sin \phi \cdot \cos 2\theta ) - c \cdot \cot \phi$$

1c $$\tau_{xy} = \tau_{yx} = \sigma \cdot \sin \phi \cdot \sin 2\theta$$

where *σ* is characteristic stress, *c* is cohesion, and *φ* is internal friction angle, *θ* is the angle between the maximum principal stress *σ*_1_ and the x-axis.

The formula of characteristic stress *σ* is introduced:

2 $$\sigma = S + c \cdot \cot \phi$$

where *S* is $\frac{{\sigma_{1} + \sigma_{3} }}{2}$, and *σ*_3_ is the minimum principal stress.

For undrained shear strength *c* > 0 and *φ* = 0, and substituting Eq. (2) into Eqs. (1a)–(1c):

3a $$\sigma_{x} = S + c \cdot \cos 2\theta$$

3b $$\sigma_{y} = S - c \cdot \cos 2\theta$$

3c $$\tau_{xy} = \tau_{yx} = c \cdot \sin 2\theta$$

The differential equations are given as follows:

4a $$\frac{{\partial \sigma_{x} }}{\partial x} + \frac{{\partial \tau_{xy} }}{\partial y} = f_{x}$$

4b $$\frac{{\partial \tau_{yx} }}{\partial x} + \frac{{\partial \sigma_{y} }}{\partial y} = f_{y}$$

where $f_{x} = \gamma \cdot k_{H}$,$f_{y} = \gamma \cdot (1 - k_{V} )$, *γ* represents the unit weight, and *k*_H_ and *k*_V_ represent the horizontal and vertical seismic coefficients. *k*_V_ = *ξ*·*k*_H_, where *ξ* is the proportional coefficient, *k*_H_ is 0 under static conditions, i.e., $f_{x} = 0$,$f_{y} = \gamma$.

Partial differential equations of pure clay seismic slip line field theory can be obtained by substituting Eqs. (3a)–(3c) into Eqs. (4a), (4b):

5a $$\frac{\partial S}{{\partial x}} - 2c\left( {\sin 2\theta \frac{\partial \theta }{{\partial x}} - \cos 2\theta \frac{\partial \theta }{{\partial y}}} \right) = f_{x}$$

5b $$\frac{\partial S}{{\partial y}} + 2c\left( {\sin 2\theta \frac{\partial \theta }{{\partial y}} + \cos 2\theta \frac{\partial \theta }{{\partial x}}} \right) = f_{y}$$

The differential equation of two families of slip lines (α and β) can be obtained according to the method of characteristic (as shown in the attachment Appendix A):

6a $$\alpha \;{\text{family}}:\;\;\left\{ \begin{gathered} \frac{{{\text{d}}y}}{{{\text{d}}x}} = {\text{tan}}\left( {\theta - \frac{\pi }{4}} \right) \hfill \\ {\text{d}}S - {2}c \cdot {\text{d}}\theta = f_{x} \cdot {\text{d}}x + f_{y} \cdot {\text{d}}y \hfill \\ \end{gathered} \right.$$

6b $$\beta \;{\text{family}}:\,\;\left\{ \begin{gathered} \frac{{{\text{d}}y}}{{{\text{d}}x}} = {\text{tan}}\left( {\theta + \frac{\pi }{4}} \right) \hfill \\ {\text{d}}S + {2}c \cdot {\text{d}}\theta = f_{x} \cdot {\text{d}}x + f_{y} \cdot {\text{d}}y \hfill \\ \end{gathered} \right.$$

**Figure 1:**
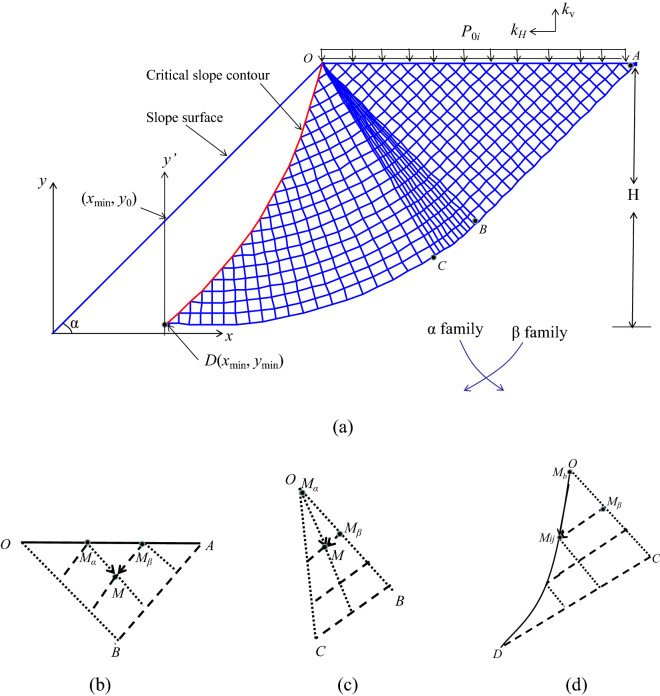
The undrained method of characteristics: (**a**) diagram of slip line field; (**b**) Cauchy boundary schematic; (**c**) degenerative Riemann boundary schematic; (**d**) mixed boundary schematic.

The finite difference method (FDM) is used to solve (6a) and (6b) approximately:

7a $$\frac{{y - y_{\alpha } }}{{x - x_{\alpha } }} = {\text{tan}}\left( {\theta_{\alpha } - \frac{\pi }{4}} \right)$$

7b $$(S - S_{\alpha } {)} - {2}c \cdot (\theta - \theta_{\alpha } ) = f_{x} (x - x_{\alpha } ) + f_{y} (y - y_{\alpha } )$$

7c $$\frac{{y - y_{\beta } }}{{x - x_{\beta } }} = {\text{tan}}\left( {\theta_{\beta } + \frac{\pi }{4}} \right)$$

7d $$(S - S_{\beta } {)} + {2}c \cdot (\theta - \theta_{\beta } ) = f_{x} (x - x_{\beta } ) + f_{y} (y - y_{\beta } )$$

where *M*_*α*_ (*x*_*α*_, *y*_*α*_, *θ*_*α*_, *S*_*α*_) and *M*_*β*_ (*x*_*β*_, *y*_*β*_, *θ*_*β*_, *S*_*β*_) are the points in the *α* and *β* families, as shown in Fig. 1, and (x, y) is the coordinate value.

The point *M* (*x*, *y*, *θ*, *S*) on the slip line is assessed using formulas (7a)–(7d), e.g., formulas (8) and (9) can be obtained by formulas (7a) and (7c), formulas (10) and (11) can be obtained by formulas (7b) and (7d):

8 $$x = \frac{{x_{\alpha } \cdot {\text{tan}}\left( {\theta_{\alpha } - \frac{\pi }{4}} \right) - x_{\beta } \cdot {\text{tan}}\left( {\theta_{\beta } + \frac{\pi }{4}} \right) - (y_{\alpha } - y_{\beta } )}}{{{\text{tan}}\left( {\theta_{\alpha } - \frac{\pi }{4}} \right) - {\text{tan}}\left( {\theta_{\beta } + \frac{\pi }{4}} \right)}}$$

9 $$\left\{ \begin{gathered} y = {(}x - x_{\alpha } ) \cdot {\text{tan}}\left( {\theta_{\alpha } - \frac{\pi }{4}} \right) + y_{\alpha } \hfill \\ y = {(}x - x_{\beta } ) \cdot {\text{tan}}\left( {\theta_{\beta } + \frac{\pi }{4}} \right) + y_{\beta } \hfill \\ \end{gathered} \right.$$

10 $$\theta = \frac{{S_{\beta } - S_{\alpha } + {2}c(\theta_{\beta } + \theta_{\alpha } ) + f_{x} (x_{\alpha } - x_{\beta } ) + f_{y} (y_{\alpha } - y_{\beta } )}}{4c}$$

11 $$S = \frac{{S_{\beta } + S_{\alpha } }}{2} + c(\theta_{\beta } - \theta_{\alpha } ) + f_{x} \left( {\frac{{2x - x_{\alpha } - x_{\beta } }}{2}} \right) + f_{y} \left( {\frac{{2y - y_{\alpha } - y_{\beta } }}{2}} \right)$$

**Figure 2:**
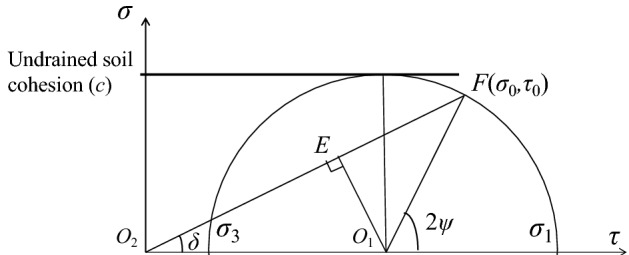
Mohr stress circle.

The normal and the shear stresses acting along the critical slope contour become zero (i.e., the stress-free boundary). Under zero-stress boundary conditions, the differential equation of the critical slope contour is $\frac{dy}{{dx}} = \tan \theta$^17^, and $S_{{{\text{ij}}}} = c$ can be obtained based on *σ*_3_ being 0 and *σ*_1_ being 2*c* in the critical slope contour (as shown in Fig. 2). The coordinate points *M*_ij_(*x*_ij_, *y*_ij_, *θ*_ij_, *S*_ij_) of seismic critical slope contour assessed by $\frac{dy}{{dx}} = \tan \theta$ in conjunction with *β* family slip line:

12 $$x_{ij} = \frac{{x_{b} \cdot {\text{tan}}\theta_{b} - x^{\prime }_{\beta } \cdot {\text{tan}}\left( {\theta^{\prime }_{\beta } + \frac{\pi }{4}} \right) - \left( {y_{b} - y^{\prime }_{\beta } } \right)}}{{{\text{tan}}\theta_{b} - {\text{tan}}\left( {\theta^{\prime }_{\beta } + \frac{\pi }{4}} \right)}}$$

13 $$\left\{ \begin{gathered} y_{ij} = {(}x - x_{b} ) \cdot {\text{tan}}\theta_{b} + y_{b} \hfill \\ y_{ij} = \left( {x - x^{\prime }_{\beta } } \right) \cdot {\text{tan}}\left( {\theta^{\prime }_{\beta } + \frac{\pi }{4}} \right) + y^{\prime }_{\beta } \hfill \\ \end{gathered} \right.$$

14 $$\theta_{ij} = \frac{{S^{\prime }_{\beta } - S_{b} + {2}c\left( {\theta^{\prime }_{\beta } + \theta_{b} } \right) + f_{x} \left( {x_{b} - x^{\prime }_{\beta } } \right) + f_{y} \left( {y_{b} - y^{\prime }_{\beta } } \right)}}{4c}$$

15 $$S_{{{\text{ij}}}} = c$$

where *M*_b_(*x*_b_, *y*_b_, *θ*_b_*, S*_b_) and *M*´_*β*_(*x*´_*β*_, *y*´_*β*_, *θ*´_*β*_, *S*´_*β*_) are known points of the seismic critical slope contour and *β* family slip line.

### Boundary value problems

There are three boundary value problems, as shown in Fig. 1b–d. The boundary value problems for a pure cohesive slope under static and seismic conditions are briefly described.

#### Seismic boundary

1. OAB Cauchy boundary.

The known points *M*_*α*_ and *M*_*β*_ at Cauchy boundary OA as shown in Fig. 1b, whose abscissa is $x = \frac{H}{\tan \alpha } + \Delta x \cdot i$, where *H* is the slope height, *α* is the slope angle, Δ*x* is the calculation step, *i* = 0 ~ *N*_1_, *N*_1_ is the step number [e.g., *N*_1_ = 3 in Fig. 1b], and the ordinate is H.

As shown in Fig. 2, $O_{1} F\sin (2\psi ) = \tau_{0}$, $O_{1} F = c$(i.e., the radius of the Mohr stress circle of undrained soil is equal to cohesion), $2\psi = 2\theta_{{\text{I}}} - \pi$, where $\tau_{0} = P_{0} \cdot k_{H}$, *P*_0_ is the load at the slope top surface, and the intersection angle (*θ*_I_) between *σ*_1_ and the x-axis can be derived as $2\theta_{{\text{I}}} - \pi = \arcsin \left( {\frac{{\tau_{0} }}{c}} \right)$:

16 $$\theta_{{\text{I}}} = \frac{\pi }{2} + \frac{1}{2}\arcsin \left( {\frac{{P_{0} \cdot k_{H} }}{c}} \right)$$

$O_{2} E = O_{2} O_{1} \sin \delta = O_{1} F\sin (2\psi - \delta )$ is also shown in Fig. 2, where $\tan (\delta ) = \frac{{\tau_{0} }}{{\sigma_{0} }}$, $\sigma_{0} = P_{0} \cdot (1 - k_{V} )$, and $\delta = \arctan \left( {\frac{{k_{H} }}{{1 - k_{V} }}} \right)$. Thus, the characteristic stress (*S*_I_ = *O*_1_*O*_2_) of *O*A is derived:

17 $$S_{{\text{I}}} = \frac{{c \cdot \sin (2\theta_{{\text{I}}} - \pi - \delta )}}{\sin \delta }$$

2. OCD Mixed boundary.

As shown in Fig. 1d, the characteristic stress (*S*_b_) of the known point *M*_b_ on the slope crest:

18 $$S_{\rm {b}} = S_{{{\text{III}}}} = c$$

According to the characteristic of the *β* family slip line integral equation (i.e.,$S + 2c\theta = const.$), the intersection angle (*θ*_III_) can be obtained:

19 $$\theta_{\rm {b}} = \theta_{{{\text{III}}}} = (S_{{\text{I}}} + 2c\theta_{{\text{I}}} - c)/2$$

3. OBC Degenerative Riemann boundary.

The known point O at the Degenerative Riemann boundary is the slope crest, as shown in Fig. 1c, and the characteristic stress is:

20 $$S_{{{\text{II}}}} = S_{{\text{I}}} + 2c(\theta_{{\text{I}}} - \theta_{{{\text{II}}}} )$$

where $\theta_{{{\text{II}}}} = \theta_{{\text{I}}} + k \cdot \frac{\Delta \theta }{{N_{2} }}$, *k* = 0 ~ *N*_2_, $\theta_{\rm {b}} = \theta_{{{\text{III}}}} = \theta_{{\text{I}}}$, *N*_2_ is the point partition of the Riemann boundary.

#### Static boundary

Boundary value problems under static conditions (i.e., *k*_H_ = *k*_V_ = 0) were given by Zhao^18^:

(1) $\theta_{{\text{I}}} = \pi /2$ and $S_{{\text{I}}} = P_{0} - c$ for OAB Cauchy boundary; (2)$\theta_{\rm {b}} = \theta_{{{\text{III}}}} = \frac{{P_{0} }}{2c} + \frac{\pi }{2} - 1$ and $S_{\rm {b}} = S_{{{\text{III}}}} = c$ for OCD Mixed boundary; (3) $\theta_{{{\text{II}}}} = \theta_{{\text{I}}} + k \cdot \frac{\Delta \theta }{{N_{2} }}$ and $S_{{{\text{II}}}} = P_{0} - c(2\theta_{{{\text{II}}}} - \pi + 1)$ for OBC Degenerative Riemann boundary, where $\Delta \theta = \theta_{{{\text{III}}}} - \theta_{{\text{I}}} = \frac{{P_{0} }}{2c} - 1$.

## Failure mechanism

The critical slope contour varies with *P*_0*i*_ for a given width (i.e., B = Δ*x*·*N*_1_ = *L*_OA_) of strip foundation at the slope top, and *P*_0*i*_ is defined:

21 $$P_{0i} = P_{0} + i \cdot \Delta P$$

where *P*_0_ is the initial load, Δ*P* is the increment of *P*_0_, and *i* = 1, 2…*n*.

**Figure 3:**
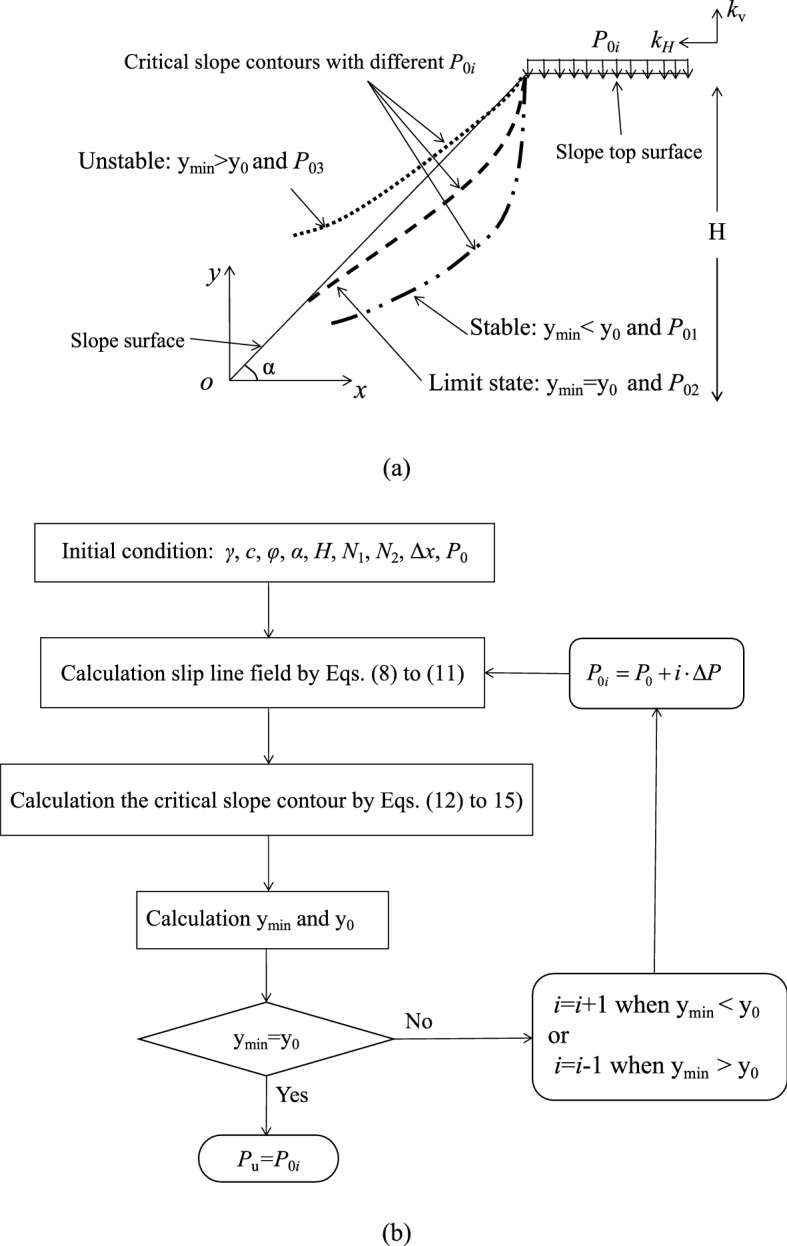
The proposed method: (**a**) failure mechanism; (**b**) calculation flow chart.

The proposed failure mechanism for predicting undrained seismic and static ultimate bearing capacity (*P*_*s*u_ or *P*_u_) of shallow strip foundation placed adjacent to the pure cohesive slope is shown in Fig. 3a: (1) the slope is in a stable state, and *P*_0*i*_ < *P*_*s*u_ or *P*_0*i*_ < *P*_u_ when the critical slope contour does not intersect with the slope (i.e., *y*_min_ < *y*_0_); (2) the slope is in a limited equilibrium state, and *P*_0*i*_ = *P*_*s*u_ or *P*_0*i*_ = *P*_u_ when *y*_min_ = *y*_0_; (3) the slope is in an unstable state, and *P*_0*i*_ > *P*_*s*u_ or *P*_0*i*_ > *P*_u_ when *y*_min_ > *y*_0_, where (*x*_min_, *y*_min_) is the minimum coordinate of the critical slope contour, and *y*_0_ = *x*_min_·tan*α*, i.e., *y'* axis is parallel to the ordinate.

It should be noted that the proposed failure mechanism is still valid when *y*_min_ < 0 or *y*_0_ < 0, and the proposed instability criterion proposed by Fang et al.^16^ is a special case of the method in this paper, i.e., *y*_min_ = *y*_0_ = 0. In addition, the right-most β slip line [i.e., the curve ABCD in Fig. 1a] is not the critical slip surface in this study. The calculation flow chart is shown in Fig. 3b. See the attachment Appendix B for the Matlab calculation program.

## Static bearing capacity

### Calculation * P*_*u*_

**Figure 4:**
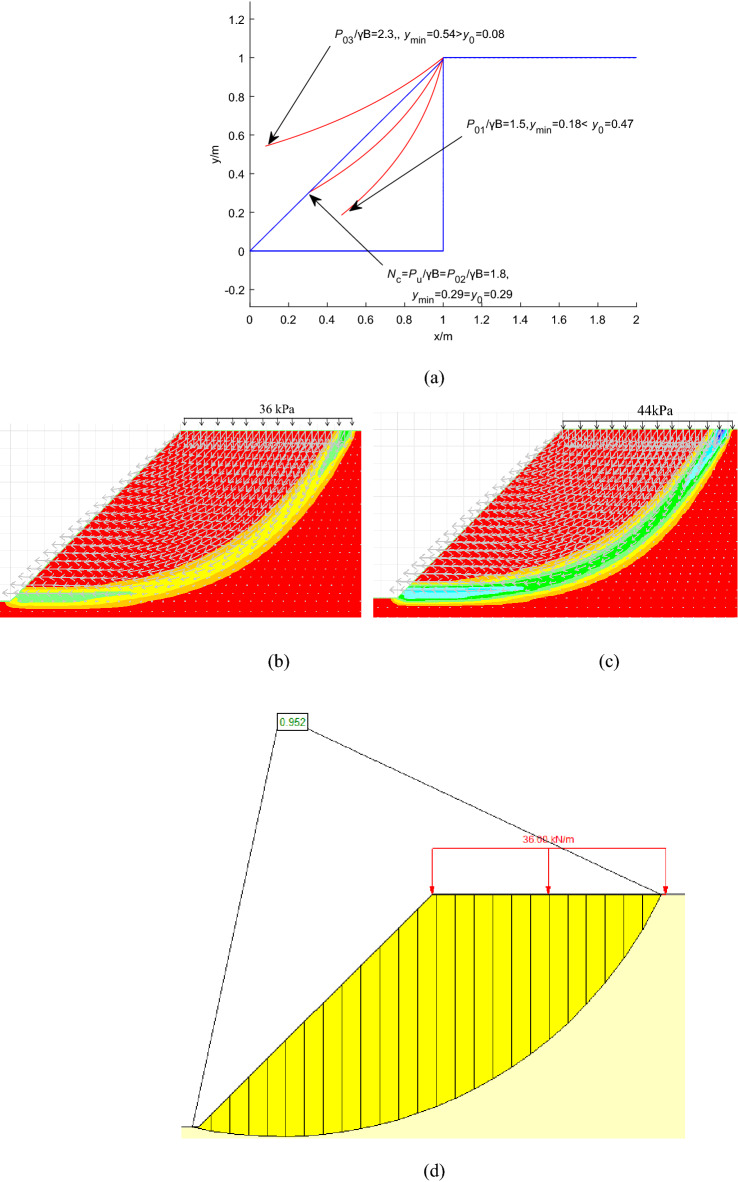

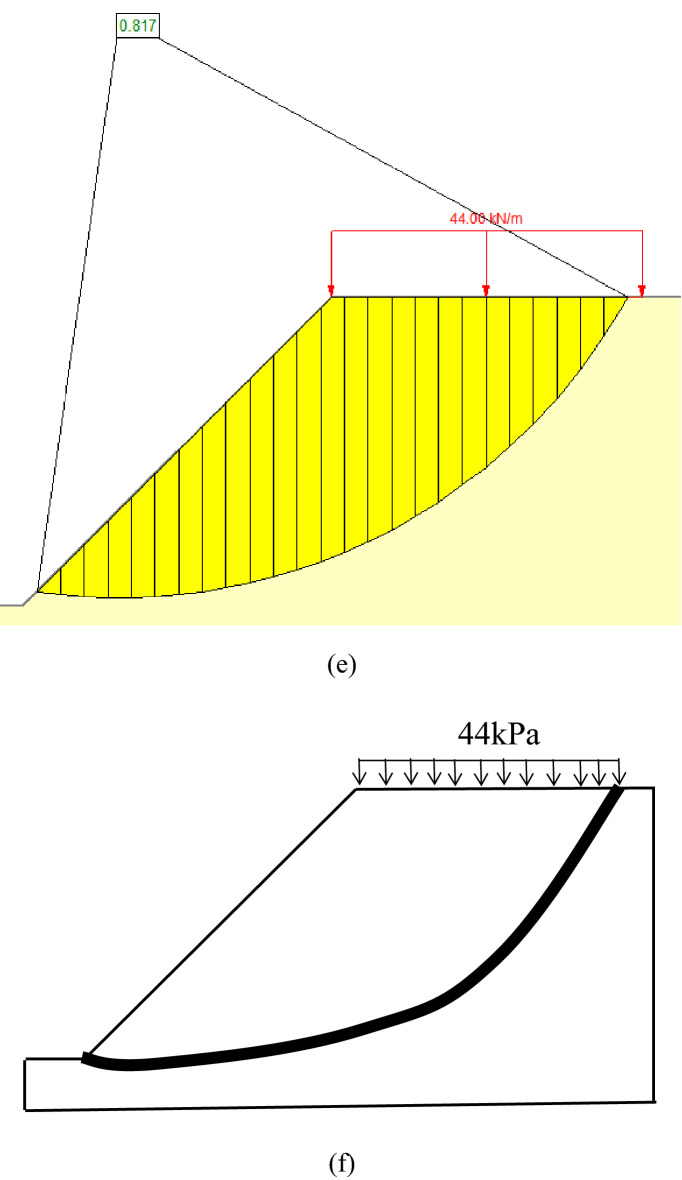
The case with c/γB = 0.6a and H/B = 1: (**a**) the proposed failure mechanism: *N*_*c*_ = 1.8 (i.e., *P*_*u*_ = 36 kPa); (**b**) the critical slip surface and FS = 0.96 using FLAC with 36 kPa; (**c**) the critical slip surface and FS = 0.85 using FLAC with 44 kPa; (**d**) the critical critical slip surface and FS = 0.95 using SLIDE with 36 kPa; (**e**) the critical critical slip surface and FS = 0.82 using SLIDE with 44 kPa; (**f**) the critical critical slip surface using FELA with 44 kPa.

The cases studied by the FELA^6^ are adopted to validate the feasibility of the proposed failure mechanism under static conditions, *γ* = 20 kN/m^3^ and the foundation width B = 1 m are constants in those cases, and the static bearing capacity factor is defined as *N*_*c*_ = *P*_*u*_/*γB*. The results assessed by the proposed failure mechanism are compared with those of LB and UB-FELA (Table 1). Table 1 shows that *N*_*c*1_ evaluated by the proposed method lies between those of LB and UB, except for the case of the strength ratio *c/γB* = 0.6^a^. The critical slip surfaces and FSs of the case of *c/γB* = 0.6^a^ obtained by the proposed method, the Strength reduction technology (FLAC), the Bishop method (SLIDE7.0), and FELA are shown in Fig. 4.

**Table 1:** Comparison of *N*_*c*_*.*

Method	*c/γB*				
	0.6^a^	1.5^a^	2^a^	2^b^	4^c^
LB	2.2	4.94	6.739	7.092	13.836
UB	2.237	5.006	6.837	7.308	14.27
This study	1.8	5	6.8	7.25	14

Note: ^a^represents H/B = 1, *α* = 45°; ^b^represents H/B = 4, *α* = 40°; ^c^represents H/B = 4, *α* = 45°.

Figure 4a shows that *y*_min_ increases with the increase of *P*_0*i*_*/γB* (*i* = 1, 2, 3), and when y_min_ = y_0_, *N*_*c*1_ = *P*_u_/*γB* = 1.8. Figure 4b and d show that FS = 0.96 and 0.95 are calculated using FLAC and SLIDE7.0 when *N*_*c*_ = 1.8 (i.e., *P*_*u*_ = 36 kPa) computed by the proposed method is imposed at the slope top. Figure 4c and e show that FS = 0.85 and 0.82 are calculated using FLAC and SLIDE7.0 when *N*_*c*_ = 2.2 (i.e., *P*_*u*_ = 44 kPa) calculated by LB-FELA is imposed at the top surface of the slope. It can be seen that the critical slip surfaces calculated by FLAC, SLIDE7.0, and FELA are almost the same when *P*_*u*_ = 44 kPa and 36 kPa are applied at the top of the slope. Therefore, the critical slip surface has been generated when *P*_*u*_ = 36 kPa is applied at the top of the slope, and the *P*_*u*_ = 44 kPa calculated by FELA overestimates the ultimate bearing capacity. According to the definition of ultimate bearing capacity, FS is 1 when the ultimate bearing capacity is imposed at the top surface of the slope. Therefore, the proposed method is more reasonable, i.e., FS = 0.96, and 0.95 are closer to 1.

### H/B varying

**Figure 5:**
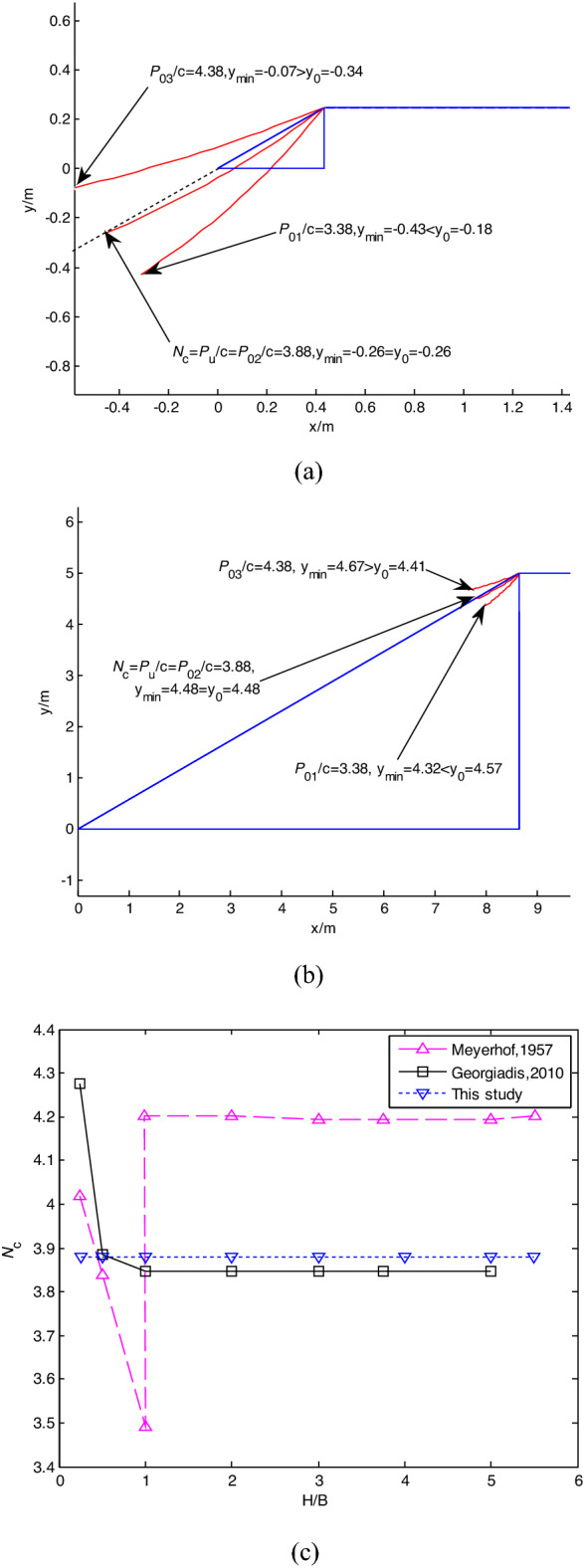
Influence H/B on *N*_*c*_ with *α* = 30° and *c/γB* = 1: (**a**) H/B = 0.25; (**b**) H/B = 5; (**c**) comparison of *N*_*c*_*.*

The cases assessed by the proposed failure mechanism, FEM (Georgiadis^4^ and Meyerhof^19^) with normalized slope height *H/B* varying from 0.25 to 5.5, *α* = 30°, *c/γB* = 1, B = 2 m are shown in Fig. 5. Figure 5a and b show that H/B does not influence on the proposed method for estimating the static bearing capacity factor. Figure 5c shows that *N*_*c*_ obtained by FEM (Georgiadis^4^ and Meyerhof^19^) decreases with the increase of H/B when H/B < 1, and *N*_*c*_ assessed by Meyerhof^19^ has an unrealistic jump when H/B = 1. *N*_*c*_ predicted by the proposed method is constant, and when H/B > 1, it is close to those of the FEM.

When *N*_*c*_ values obtained by the proposed method, Meyerhof^19^ and FEM are imposed at the slope top, FSs calculated by the Bishop method with SLIDE7.0 are shown in Table 2. Table 2 shows that *N*_*c*_ and FS computed by Meyerhof^19^ are unstable, but *N*_*c*_ calculated by the proposed method are close to those of FEM, and FS_TS_ is close to 1.0 for each H/B case.

**Table 2:** Comparison of *N*_*c*_ and *FS.*

Methods		H/B				
		0.25	0.5	1	3	5
Meyerhof (1957)	*N*_*c*_	4	3.84	3.5/4.2	4.2	4.2
	*FS*	1.035	1.042	1.115/0.946	0.953	0.958
Georgiadis(2010)	*N*_*c*_	4.27	3.88	3.85	3.85	3.85
	*FS*	0.972	1.032	1.024	1.031	1.038
This study	*N*_*c*TS_	3.88	3.88	3.88	3.88	3.88
	*FS*_TS_	1.066	1.032	1.016	1.024	1.03

Note: Symbol “/” represents *N*_*c*_ and *FS* oscillating; TS represents This study.

### c/γB and α varying

**Figure 6:**
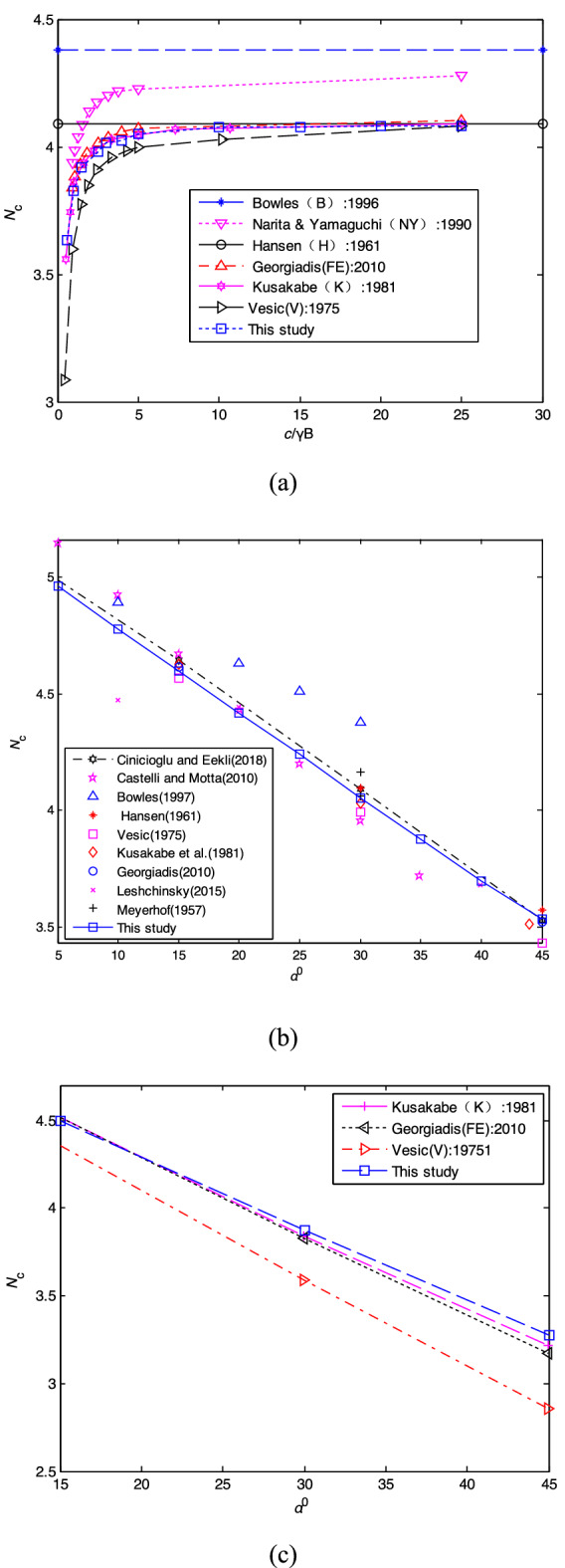
Variation of *N*_c_ with *c*_*u*_*/γB* and *α*: (**a**) *c/γB* varying, *α* = 30°; (**b**) *α* varying, *c/γB* = 5; (**c**) *α* varying, *c/γB* = 1.

The cases with *c/γB* varying from 0.5 to 25 and *α* = 30° were assessed by the proposed method and other researchers, as shown in Fig. 6a. The cases with *α* varying from 5° to 45° and *c/γB* = 5 were assessed by the proposed method and other researchers, as shown in Fig. 6b. The cases with *α* varying from 15° to 45° and *c/γB* = 1 were assessed by the proposed method and other researchers, as shown in Fig. 6c.

Figure 6a shows that *N*_*c*_ assessed by the proposed failure mechanism is in good agreement with those of FEM (Georgiadis^4^) and UB (Kusakabe et al.^20^), and *N*_*c*_ increases with the increase of *c/γB*. Compared with FEM and UB, *N*_*c*_ assessed by the empirical equations of Hansen (H) (1961)^21^ and Bowles (B) (1996)^22^ is unreasonable since *N*_*c*_ is constant. *N*_*c*_ assessed by the empirical equation of Vesic (V) (1975)^23^ or an assumed log-spiral failure mechanism of Narita and Yamaguchi (NY) (1990)^24^ is underestimated or overestimated.

Figure 6b shows that *N*_*c*_ assessed by the proposed failure mechanism is close to those of FEM (Georgiadis^4^; Cinicioglu and Erkli^5^) and UB (Kusakabe et al.^20^). Castelli and Motta^1^ used the limit equilibrium method of slices and assumed circular failure surfaces, which caused the deviation of *N*_*c*_. *N*_*c*_ was overestimated and underestimated by Bowles’s^22^ solution and discontinuity layout optimization (Leshchinsky^11^). Figure 6c shows that *N*_*c*_ assessed by the proposed failure mechanism is close to those of FEM and UB, and Vesic (V) (1975)’s^23^ solution underestimates *N*_*c*_.

Figures 4a, 5a and b show that the position of the critical slope contour and the slope surface change with the increase of load. When the load on the top of the slope increases, the position of the critical slope contour transfers from the inside of the slope to the outside of the slope. The new limit evaluation index (i.e., y_min_ = y_0_) can be used to evaluate the ultimate bearing capacity, which solves the problem of overestimating the ultimate bearing capacity in limit analysis when the cohesion is small. When the aspect ratio changes, the ultimate bearing capacity calculated by the current method is oscillating, and the proposed limit evaluation index gives a stationary solution.

## Seismic bearing capacity

### Numerical convergence of *P*_*su*_

**Figure 7:**
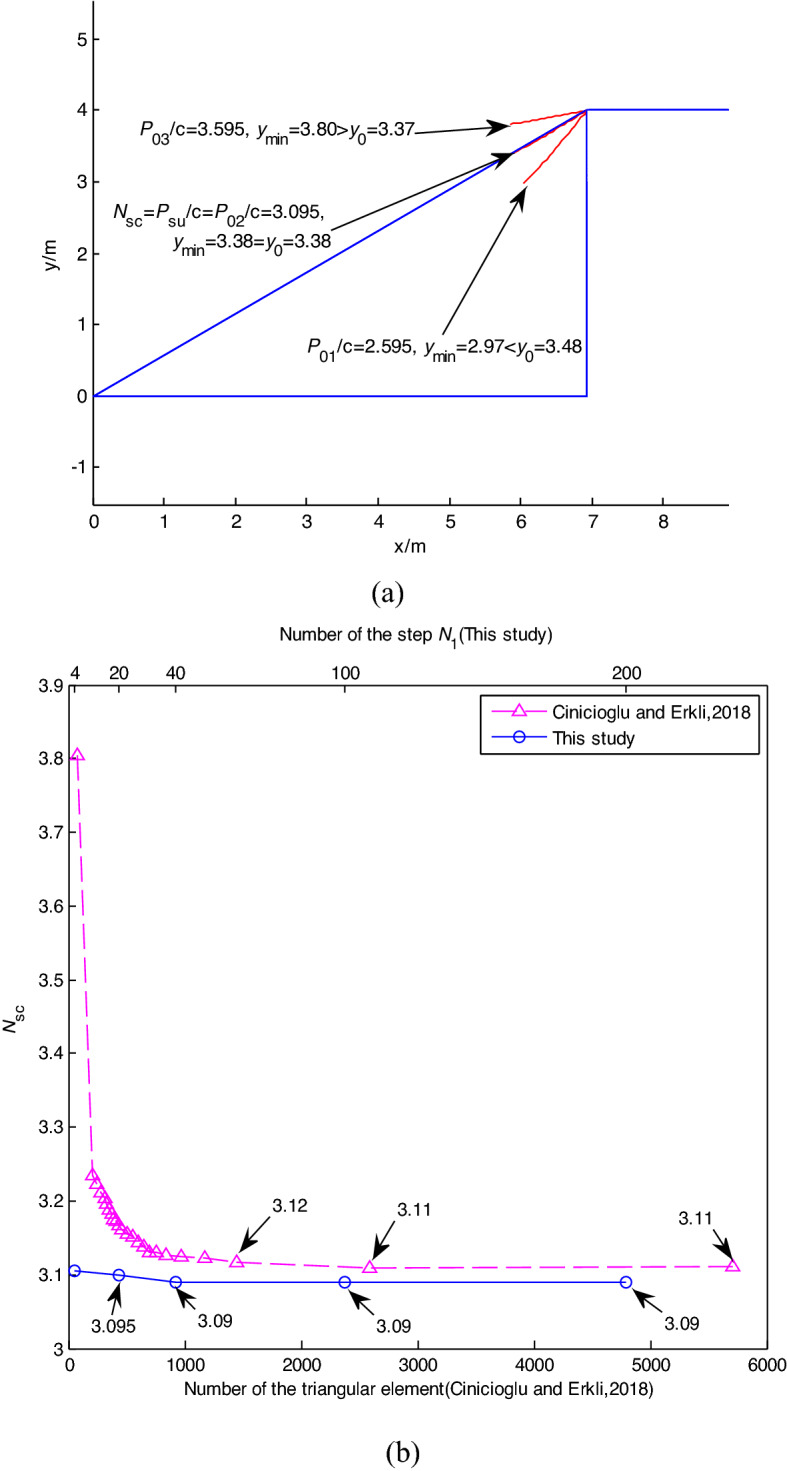
The proposed failure mechanism undern the seismic condition: (**a**) calculation with B = 2 m, *N*_1_ = 20, Δ*x* = 0.1, *k*_H_ = 0.2; (**b**) numerical convergence.

The case with *γ* = 20 kN/m^3^, *c* = 100 kPa, *H* = 4 m, *B* = 2 m, *α* = 30°, and *k*_H_ = 0.2 is used to validate the convergence of the proposed failure mechanism. The seismic bearing capacity factor defined as *N*_*sc*_ = *P*_*s*u_/*c* assessed by the proposed method with *N*_1_ = 20 is 3.095, as shown in Fig. 7a. It can be seen that y_min_ increases with the increase of *P*_0*i*_*/c* (*i* = 1, 2, 3), *P*_*s*u_ = *P*_02_ = 309.5 kPa when y_min_ = y_0_.

Generally, a larger step number *N*_1_ in FDM or a higher number of triangular elements in FEM can achieve a more accurate result, as shown in Fig. 7b. Figure 7b reveals that *N*_*sc*_ assessed by the proposed method has converged quite well to the limit value (3.09). The limit value, i.e., *N*_*sc*_ = 3.09 and 3.11 obtained by the proposed method and FEM^5^, has a minor difference with an error of 0.6%.

### The influence of *k*_*H*_ and H/B

**Figure 8:**
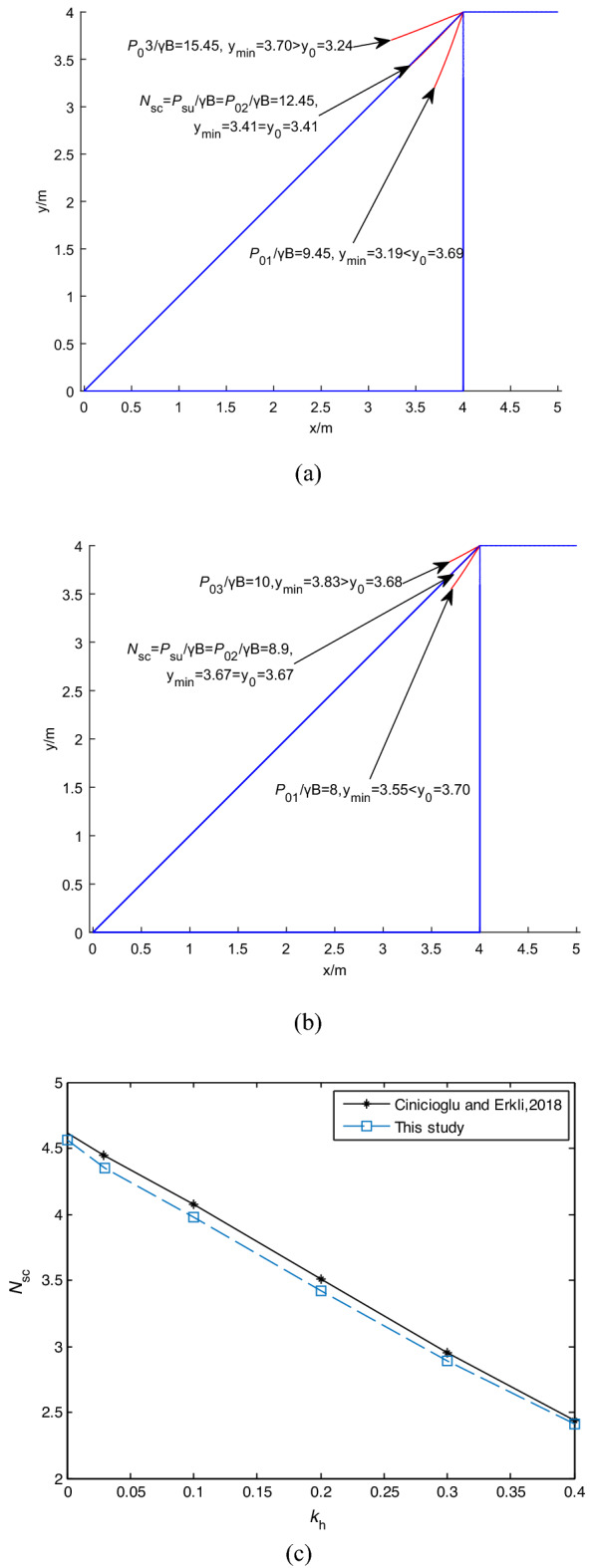

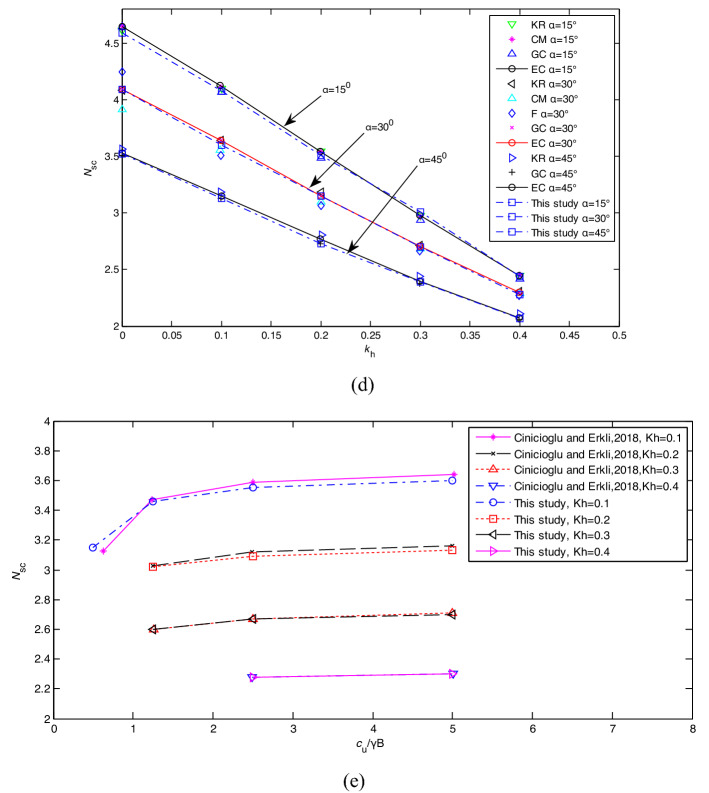
The influence of *k*_H_ on *N*_*sc*_ = *P*_*s*u_/*c*: (**a**) *k*_H_ = 0.1; (**b**) *k*_H_ = 0.35; (**c**) H/B = 1, 2, and 4; (**d**) *α* varying; (e) *c/γB* varying.

*N*_*sc*_ = *P*_*su*_/*γB* defined by Keshavarz et al.^6^ were assessed with *α* = 45°, H/B = 4, *c/γB* = 4.0, *k*_H_ = 0.1, 0.15, 0.3, 0.35 by LB and UB -FELA, and the proposed failure mechanism as shown in Table 3. The results of the proposed failure mechanism with *k*_H_ = 0.1, 0.35 are shown in Fig. 8a and b. Table 3 indicates that *N*_*sc*1_ assessed by the proposed method lies between those of LB and UB-FELA, with the maximum relative difference being 2.8%. Figure 8a shows that the rule of the proposed method is still valid when *k*_H_ varies, i.e., y_min_ increases with the increase of *P*_0*i*_/*γB*.

**Table 3:** The influence of *k*_H_ on *N*_*sc*_ = *P*_*su*_/(*γB*).

Methods	*k*_H_			
	0.1	0.15	0.3	0.35
LB	12.38	11.63	9.483	8.837
UB	12.785	12.01	9.777	9.091
This study	12.45	11.75	9.5	8.9

The cases with *α* = 15°, H/B = 1, 2, 4, *c/γB* = 2.5, and *k*_H_ = 0, 0.1, 0.2, 0.3, 0.4 are analyzed using the proposed method and FEM^5^ as shown in Fig. 8c. Figure 8c shows that *N*_*sc*_ values assessed by the proposed method and FEM have minor differences, with the maximum error being 1.3%. Table 3 and Fig. 8c show that *N*_*sc*_ = *P*_*s*u_/*c* and *N*_*sc*1_ = *P*_*su*_/*γB* assessed by the proposed method, LB and UB-FELA and FEM decrease with *k*_H_ increasing.

### The influence of α and c/γB

The cases with *α* = 15°, 30°, 45°, H/B = 1, 2, 4, *c/γB* = 5, and *k*_H_ varying from 0 to 0.4 assessed by the proposed method, FEM (EC), the current method of characteristics (KR), the limit equilibrium method of slices (CM), the finite element lower bound method (F), and the upper bound method (GC) are shown in Fig. 8d. It shows that *N*_*sc*_ decreases with the increase of *k*_H_, *α*. *N*_*sc*_ values assessed by the proposed method are close to those obtained by the other methods.

The cases with *k*_H_ varying from 0.1 to 0.4, *c/γB* varying from 0.5 to 5, *α* = 30°, and H/B = 1 assessed by the proposed method and FEM are shown in Fig. 8e. Figure 8e reveals that *N*_*sc*_ assessed by the proposed method are close to those of FEM, and *N*_*sc*_ decreases with the increase of *k*_H_.

Figures 7a, 8a and b show that the change rule of the relationship position between the critical slope contour and the slope surface under seismic conditions is consistent with the static condition. With the increase of the load on the top of the slope, the position of the critical slope contour changes from inside to outside of the slope. Compared with FEM, the proposed limit evaluation index converges faster. The calculation results are consistent with the current methods.

## Conclusions

The seismic or static undrained slip line field theory of the pure cohesive slope was derived. The Cauchy, Riemann, and Mixed boundary value problems of the undrained slope were given. The critical slope contour can be obtained by the undrained slip line field theory. A new failure mechanism was proposed for assessing the seismic and static undrained ultimate bearing capacity, i.e., when the minimum ordinate (*y*_min_) in the undrained critical slope contour is equal to the ordinate (*y*_0_) of the slope surface, the load is the ultimate bearing capacity.

When the strength ratio is small, the FELA overestimates the undrained ultimate bearing capacity. H/B does not affect the proposed method for evaluating static and seismic undrained ultimate bearing capacity. The undrained ultimate bearing capacity decreases with *α* increasing and *c/γB* decreasing. The convergence of the proposed failure mechanism under the seismic conditions is proved by comparing it with FEM, and the seismic undrained bearing capacity decreases with the increase of *k*_H_. The proposed method is more reasonable because the FS is closer to 1 when the undrained ultimate bearing capacity evaluated by the proposed method is applied to the slope top surface.

The proposed failure mechanism does not require prior assumption or search for failure modes, and a new limit state evaluation index is given for evaluating the undrained ultimate bearing capacity. For the conditions of the water pressure and heterogeneity, this method needs further study.

## Supplementary Information

Supplementary Information.

## Data Availability

All data generated or analysed during this study are included in this published article [and its supplementary information files].

## Abbreviations

*c*: Cohesion
$f_{x}$, $f_{y}$: Horizontal and vertical seismic forces
*B*: Foundation width
*H*: Slope height
*i*, *j*: Natural numbers
*k*_*H*_, *k*_*V*_: Horizontal and vertical seismic coefficients
*M**, M*_*α*_, *M*_*β*_, *M*´_*β*_: Points on a slip line
*M*_*b*,_*M*_*ij*_: Points on the critical slope contour
*N*_1_, *N*_2_: Number of calculation steps in Cauchy and Riemann boundary
*N*_c_, *N*_Sc_: Static and seismic bearing capacity factors
*P*_0_, Δ*P*: Load and increment load on the top of the slope
*P*_*u*_*, P*_*su*_: Static and seismic ultimate bearing capacities
*S*_*b*_*, S*_*ij*_, *S*_*α*_, *S*_*β*_, *S*´_*β*_, *S*_I_, *S*_II_, *S*_III_: Mean value of principal stress
*x*_min_*, x*_*b*_ , *x*_*ij*_ , *x*_*α*_, *x*_*β*_, *x*´_*β*_: Abscissa values
*x*: Calculation step
*y*_0_*, y*_Min_, *y*_b_, *y*_*ij*_ , *y*_*α*_ , *y*_*β*_ , *y*´_*β*_: Ordinate values
*α*: Alpha family slip line or slope angle
*β*: Beta family slip line
*γ*: Unit weight
*θ**, θ*_*b*_ , *θ*_*ij*_ , *θ*_*α*_, *θ*_*β*_, *θ*´_*β*_, *θ*_I_, *θ*_II_, *θ*_III_: Intersection angles between maximum principal stress and x axis
*μ*: Mean angle between two family slip lines
$$\xi$$: Proportional coefficient
*σ*: Characteristic stresses
*σ*_*x*_*, σ*_*y*_: Normal stresses
*σ*_1_*, σ*_3_: Maximum and minimum principal stresses
*τ*_*x*_*, τ*_*y*_: Shear stresses
*φ*: Internal friction angle
