# Supplementary material for: New failure mechanism for evaluating seismic and static undrained bearing capacity adjacent to 2D slope
Source: Sci Rep. 2023 Jan 31;13:1712. doi: 10.1038/s41598-023-28415-4 (PMC9889396; doi:10.1038/s41598-023-28415-4)
Supplement: Supplementary file 1 — Supplementary Information. [file 41598_2023_28415_MOESM1_ESM.pdf]

Appendix A

Partial differential equations of pure clay seismic slip line field theory:

$$\frac{\partial S}{\partial x}-2c(\sin 2\theta\frac{\partial \theta}{\partial x}-\cos 2\theta\frac{\partial \theta}{\partial y})=f_x \tag{1a}$$

$$\frac{\partial S}{\partial y}+2c(\sin 2\theta\frac{\partial \theta}{\partial y}+\cos 2\theta\frac{\partial \theta}{\partial x})=f_y \tag{1b}$$

Formula (1a) multiplied by $\cos(\theta\mp\frac{\pi}{4})$, and formula (1b) multiplied by $\sin(\theta\mp\frac{\pi}{4})$ can get:

$$\frac{\partial S}{\partial x}\cdot\cos(\theta\mp\frac{\pi}{4})-2c(\sin 2\theta\frac{\partial \theta}{\partial x}-\cos 2\theta\frac{\partial \theta}{\partial y})\cdot\cos(\theta\mp\frac{\pi}{4})-f_x\cdot\cos(\theta\mp\frac{\pi}{4})=0 \tag{2a}$$

$$\frac{\partial S}{\partial y}\cdot\sin(\theta\mp\frac{\pi}{4})+2c(\cos 2\theta\frac{\partial \theta}{\partial x}+\sin 2\theta\frac{\partial \theta}{\partial y})\cdot\sin(\theta\mp\frac{\pi}{4})-f_y\cdot\sin(\theta\mp\frac{\pi}{4})=0 \tag{2b}$$

Another form of equation (2c) can be obtained by formula adding Eq. (2a) to Eq. (2b):

$$(\frac{\partial S}{\partial x}\mp 2c\cdot\frac{\partial \theta}{\partial x}-f_x)\cos(\theta\mp\frac{\pi}{4})+(\frac{\partial S}{\partial y}\mp 2c\cdot\frac{\partial \theta}{\partial y}-f_y)\sin(\theta\mp\frac{\pi}{4})=0 \tag{2c}$$

The following equations can be obtained by multiplying Eq (2c) by d*x* and d*y*:

$$(\frac{\partial S}{\partial x}\mp 2c\cdot\frac{\partial \theta}{\partial x}-f_x)\cos(\theta\mp\frac{\pi}{4})dx+(\frac{\partial S}{\partial y}\mp 2c\cdot\frac{\partial \theta}{\partial y}-f_y)\sin(\theta\mp\frac{\pi}{4})dx=0 \tag{2d}$$

$$(\frac{\partial S}{\partial x}\mp 2c\cdot\frac{\partial \theta}{\partial x}-f_x)\cos(\theta\mp\frac{\pi}{4})dy+(\frac{\partial S}{\partial y}\mp 2c\cdot\frac{\partial \theta}{\partial y}-f_y)\sin(\theta\mp\frac{\pi}{4})dy=0 \tag{2e}$$

Transforming the Eq (2d) and (2e):

$$(\frac{\partial S}{\partial x}\mp 2c\cdot\frac{\partial \theta}{\partial x}-f_x)dx=Z_1\tan(\theta\mp\frac{\pi}{4})dx \tag{2f}$$

$$(\frac{\partial S}{\partial y}\mp 2c\cdot\frac{\partial \theta}{\partial y}-f_y)dy=Z_2\cot(\theta\mp\frac{\pi}{4})dy \tag{2g}$$

where $Z_1=-(\frac{\partial S}{\partial y}\mp 2c\cdot\frac{\partial \theta}{\partial y}-f_y)$, $Z_2=-(\frac{\partial S}{\partial x}\mp 2c\cdot\frac{\partial \theta}{\partial x}-f_x)$.

According to spatial total differential equations: $dS=\frac{\partial S}{\partial x}dx+\frac{\partial S}{\partial y}dy$ and $d\theta=\frac{\partial \theta}{\partial x}dx+\frac{\partial \theta}{\partial y}dy$, and adding Eq.(2f) to Eq.(2g):

$$dS\mp 2c\cdot d\theta-f_xdx-f_ydy=Z_1\tan(\theta\mp\frac{\pi}{4})dx+Z_2\cot(\theta\mp\frac{\pi}{4})dy \tag{3a}$$

The following equations can be obtained by multiplying Eq. (3a) by $\sin(\theta\mp\frac{\pi}{4})$ and $\cos(\theta\mp\frac{\pi}{4})$:

$$(dS\mp 2c\cdot d\theta-f_xdx-f_ydy)\sin(\theta\mp\frac{\pi}{4})=Z_1\tan(\theta\mp\frac{\pi}{4})\sin(\theta\mp\frac{\pi}{4})dx+Z_2\cos(\theta\mp\frac{\pi}{4})dy \tag{3b}$$

$$(dS\mp 2c\cdot d\theta-f_xdx-f_ydy)\cos(\theta\mp\frac{\pi}{4})=Z_1\sin(\theta\mp\frac{\pi}{4})dx+Z_2\cot(\theta\mp\frac{\pi}{4})\cos(\theta\mp\frac{\pi}{4})dy \tag{3c}$$

Substituting Eq.(2f) into Eq.(3b), and substituting Eq.(2g) into Eq.(3c):

$$(dS \mp 2c \cdot d\theta - f_x dx - f_y dy)\sin(\theta \mp \frac{\pi}{4}) = -Z_2 \sin(\theta \mp \frac{\pi}{4})dx + Z_2 \cos(\theta \mp \frac{\pi}{4})dy \tag{3d}$$

$$(dS \mp 2c \cdot d\theta - f_x dx - f_y dy)\cos(\theta \mp \frac{\pi}{4}) = Z_1 \sin(\theta \mp \frac{\pi}{4})dx - Z_1 \cos(\theta \mp \frac{\pi}{4})dy \tag{3e}$$

Formulas (3d) and (3e) are transformed into:

$$Z_2 = \frac{dS \mp 2c \cdot d\theta - f_x dx - f_y dy}{\cos(\theta \mp \frac{\pi}{4})dy - \sin(\theta \mp \frac{\pi}{4})dx}\sin(\theta \mp \frac{\pi}{4}) \tag{4a}$$

$$Z_1 = \frac{dS \mp 2c \cdot d\theta - f_x dx - f_y dy}{\sin(\theta \mp \frac{\pi}{4})dx - \cos(\theta \mp \frac{\pi}{4})dy}\cos(\theta \mp \frac{\pi}{4}) \tag{4b}$$

The differential equation of two families of slip lines can be obtained according to the method of characteristic:

$$\begin{cases} \dfrac{\mathrm{d}y}{\mathrm{d}x} = \tan(\theta - \dfrac{\pi}{4}) \\ \mathrm{d}S - 2c \cdot \mathrm{d}\theta = f_x \cdot \mathrm{d}x + f_y \cdot \mathrm{d}y \end{cases} \tag{5a}$$

$$\begin{cases} \dfrac{\mathrm{d}y}{\mathrm{d}x} = \tan(\theta + \dfrac{\pi}{4}) \\ \mathrm{d}S + 2c \cdot \mathrm{d}\theta = f_x \cdot \mathrm{d}x + f_y \cdot \mathrm{d}y \end{cases} \tag{5b}$$

Appendix B

%%Matlab calculation program

%%Main program

```
MaxPoint = 2000;
MaxValue = 2000;
for i=1:1:MaxPoint
    for j=1:1:MaxPoint
      PointValue{i,j} = [MaxValue,MaxValue,MaxValue,0,0,0];
    end
end
tic
% Enter initial value
Gamma=20;                                           % Unit weight
C0=80;                                              % cohesion
Alpha0=(45/180*pi);                                   % slope angl
H=4;                                                % slope height
kh=-0.1
xi=0.0;
kv=xi*(abs(kh))    ;
delta=atan(abs(kh)/(1-kv)) ;
delta0=delta/pi*180;
F=1   ;
C1=C0/F     ;
Alpha1=(Alpha0/pi*180) ;
BuchangX=0.01;                                        % calculation step
N1=100;                                              % step number
N2=10;                                              % the point partition of the
Riemann boundary
B=BuchangX*N1                                         % foundation width
P1=36                                               % load
P=P1/(Gamma*B)
X_3=-H/tan(Alpha0);
Y_3=H;
Y_3_0 =H ;
X_3_2_1=0;
Y_3_2_1=0;
Y_3_2_1_0 = 0;
Mu=pi/4;
Point_0_0 = {0,0,0,0,0,0};
```

```
 Count1 = (N1+1)*(N1+2)/2;
 Count2 = (N1+1)*N2;
 Count3 = N1*(N1+1)/2 ;
 Count = Count1+Count2+Count3;
 %%Cauchy boundary
Sigma0=P1*(1-kv)     ;
tau0=P1*(abs(kh))     ;
Theta1 = pi/2+(1/2)*asin(tau0/C1)   ;
Sigma1 =(C1*sin(2*Theta1-pi-delta))/sin(delta)     ;
        I=0;
       for i=1:1:(N1+1)
           for j=i:-1:1
                 if(j==i)
                  PointValue{i,j} = [X_3_2_1+(N1+1-i)*BuchangX, Y_3_2_1,
Y_3_2_1,Theta1,Sigma1,Sigma1];
                  I=I+1;
                  Point{I} = [i,j];
                 else
                  p1=PointValue{i,j+1};
                  p2=PointValue{i-1,j};
                  x1 = p1(1);
                  y1 = p1(2);
                  o1 = p1(4);
                  q1 = p1(5);
                  x2 = p2(1);
                  y2 = p2(2);
                  o2 = p2(4);
                  q2 = p2(5);
                  dd=callfun(x1,y1,o1,q1,x2,y2,o2,q2,Mu,C1,Gamma,kh,kv);
                  PointValue{i,j} = [dd(1), dd(2),
dd(3),dd(4),dd(5),dd(6)];
                  I=I+1;
                  Point{I} = [i,j];
                 end
            end
       end
%%Degenerative Riemann boundary
DetaXita=(Sigma1-C1)/(2*C1);

       for i=(N1+1+1):1:(N1+1+N2)
           ii = i-(N1+1);
```

```
            Theta2=Theta1+ii*DetaXita/N2;
             Sigma2=Sigma1+2*C1*(Theta1-Theta2);

            for j=(1+N1):-1:1
                  if(j==(1+N1))
                    PointValue{i,j} = [0, 0, 0,Theta2,Sigma2,Sigma2];
                    I=I+1;
                    Point{I} = [i,j];
                  else

                    p1=PointValue{i,j+1};
                    p2=PointValue{i-1,j};
                    x1 = p1(1);
                    y1 = p1(2);
                    o1 = p1(4);
                    q1 = p1(5);
                    x2 = p2(1);
                    y2 = p2(2);
                    o2 = p2(4);
                    q2 = p2(5);
                    dd=callfun(x1,y1,o1,q1,x2,y2,o2,q2,Mu,C1,Gamma,kh,kv);
                    PointValue{i,j} = [dd(1), dd(2),
dd(3),dd(4),dd(5),dd(6)];
                      I=I+1;
                    Point{I} = [i,j];

                  end
            end
        end

 %% Mixed boundary
        Sigma3 = C1;
        for i=(N1+1+N2+1):1:(N1+1+N2+N1)
            for j=(N1+1+N2+N1+1-i):-1:1
                  if(j==(N1+1+N2+N1+1-i))
                    p1=PointValue{i-1,j+1};
                    p2=PointValue{i-1,j};
                    x1 = p1(1);
                    y1 = p1(2);
                    o1 = p1(4);
                    q1 = p1(5);
```

```
                x2 = p2(1);
                y2 = p2(2);
                o2 = p2(4);
                q2 = p2(5);
                dd=callfan(x1,y1,o1,q1,x2,y2,o2,q2,Mu,C1,Gamma,kh,kv);
%% The calculation function of the critical slope contour
                PointValue{i,j} = [dd(1), dd(2),
dd(3),dd(4),Sigma3,Sigma3];
                I=I+1 ;
                Point{I} = [i,j];
               else
                p1=PointValue{i,j+1};
                p2=PointValue{i-1,j};
                x1 = p1(1);
                y1 = p1(2);
                o1 = p1(4);
                q1 = p1(5);
                x2 = p2(1);
                y2 = p2(2);
                o2 = p2(4);
                q2 = p2(5);
                dd=callfun(x1,y1,o1,q1,x2,y2,o2,q2,Mu,C1,Gamma,kh,kv);
                PointValue{i,j} = [dd(1), dd(2),
dd(3),dd(4),dd(5),dd(6)];
                I=I+1;
                Point{I} = [i,j];
               end
          end
       end

for k=1:1:I
    i=Point{k}(1);
    j=Point{k}(2);

    p=PointValue{i,j};

    p(1) = p(1)-X_3;
    p(2) = Y_3-p(2);
    p(3) = Y_3-p(3);
    PointValue{i,j}=[p(1), p(2), p(2),p(4),p(5),p(6)];
```

```
end
Y_3_0 = Y_3 - Y_3_0;
Y_3_2_1_0 = Y_3 - Y_3_2_1_0;
CountAlpha =2*N1+N2+1;
 CountBeta =N1+1;
%%Alpha lines
for i=1:1:CountAlpha
    UN_0 = 0;
    for j=1:1:CountBeta
      p=PointValue{i,j};
      if p(1)~=MaxValue || p(2)~=MaxValue
          UN_0 = UN_0+1;
      end
    end
    x_p = zeros(1,UN_0);
    y_p = zeros(1,UN_0);
    UN_0 = 0;
    for j=1:1:CountBeta
      p=PointValue{i,j};
      if p(1)~=MaxValue || p(2)~=MaxValue
          UN_0 = UN_0+1;
          x_p(1,UN_0) = p(1);
          y_p(1,UN_0) = p(2);
      end
    end
    hold on
   % plot(x_p,y_p,'w')
 plot(x_p,y_p)
end
%%Beta lines
for j=1:1:CountBeta
    UN_0 = 0;
    for i=1:1:CountAlpha
      p=PointValue{i,j};
      if p(1)~=MaxValue || p(2)~=MaxValue
          UN_0 = UN_0+1;
      end
    end
    x_p = zeros(1,UN_0);
    y_p = zeros(1,UN_0);
    UN_0 = 0;
```

```
    for i=1:1:CountAlpha
     p=PointValue{i,j};
     if p(1)~=MaxValue || p(2)~=MaxValue
         UN_0 = UN_0+1;
         x_p(1,UN_0) = p(1);
         y_p(1,UN_0) = p(2);
     end
    end
   hold on
  %  plot(x_p,y_p,'w')
 plot(x_p,y_p)
end
Y_3_0;
Y_3_2_1_0;
UN_0 = 0;
for j=1:1:CountBeta
    for i=1:1:CountAlpha
     p=PointValue{i,j};
     if  p(2)==Y_3_2_1_0
         UN_0 = UN_0+1;
     end
    end
end
x_p = zeros(1,UN_0);
y_p = zeros(1,UN_0);
UN_0 = 0;
for j=1:1:CountBeta
    for i=1:1:CountAlpha
     p=PointValue{i,j};
     if  p(2)==Y_3_2_1_0
         UN_0 = UN_0+1;
         x_p(1,UN_0) = p(1);
         y_p(1,UN_0) = p(2);

     end
    end
end
hold on
plot(x_p,y_p,'b')
%%slope surface
X_3=0;
```

```
Y_3_0 =0;
X_3_2_1=H/tan(Alpha0);
Y_3_2_1_0=H;
x_po = zeros(1,2);
y_po = zeros(1,2);
x_po(1,1)=X_3;
y_po(1,1) = Y_3_0;
x_po(1,2)=X_3_2_1;
y_po(1,2)=Y_3_2_1_0;
hold on
plot(x_po,y_po,'b')
%%critical slope contour
x_xie = zeros(1,CountBeta);
y_xie = zeros(1,CountBeta);
UN_0 = 0;
for j=1:1:CountBeta
    for i=1:1:CountAlpha
      p=PointValue{i,j};
      if p(1)~=MaxValue || p(2)~=MaxValue
        x_xie(1,j)=p(1);
        y_xie(1,j) = p(2);
      end
    end
end
hold on
plot(x_xie,y_xie,'r')
x_sj = zeros(1,3);
y_sj = zeros(1,3);
x_sj(1,1) = 0;
y_sj(1,1) = 0;
x_sj(1,2) = H/tan(Alpha0);
y_sj(1,2) = 0;
x_sj(1,3) = H/tan(Alpha0);
y_sj(1,3) = H;
hold on
plot(x_sj,y_sj,'b')
xlabel('x/m'); ylabel('y/m');
axis equal;
xmin=PointValue{2*N1+N2+1,1}(1);
ymin=PointValue{2*N1+N2+1,1}(2)
```

```
y0=tan(Alpha0)*(xmin)     %% y0<ymin: unstable;  y0=ymin: limit state;
y0>ymin: stable
toc
```

%% slip lines program

```
function dd=callfun(x1,y1,o1,p1,x2,y2,o2,p2,u,c,r,kh,kv)
   dd(1)=(x1*tan(o1-u)-x2*tan(o2+u)-(y1-y2))/(tan(o1-u)-tan(o2+u));
   dd(2)=(dd(1)-x1)*tan(o1-u)+y1;
   dd(3)=(dd(1)-x2)*tan(o2+u)+y2;
   dd(4)=(r*kh*(x1-x2)+r*(1-kv)*(y1-y2)+(p2-p1)+2*c*(o2+o1))/(4*c);

dd(5)=r*kh*(dd(1)-x1/2-x2/2)+r*(1-kv)*(dd(2)-y1/2-y2/2)+(p1+p2)/2+c*(o2
-o1);

dd(6)=r*kh*(dd(1)-x1/2-x2/2)+r*(1-kv)*(dd(2)-y1/2-y2/2)+(p1+p2)/2+c*(o2
-o1);
end
```

%% critical slope contour program

```
function dd=callfan(x1,y1,o1,p1,x2,y2,o2,p2,u,c,r,kh,kv)
   dd(1)=(x1*tan(o1)-x2*tan(o2+u)-(y1-y2))/(tan(o1)-tan(o2+u));
   dd(2)=(dd(1)-x1)*tan(o1)+y1;
   dd(3)=(dd(1)-x2)*tan(o2+u)+y2;
   dd(4)=(r*kh*(x1-x2)+r*(1-kv)*(y1-y2)+(p2-p1)+2*c*(o2+o1))/(4*c);
end
```
